# Neutralizing Antibody Response to Hepatitis C Virus

**DOI:** 10.3390/v3112127

**Published:** 2011-11-02

**Authors:** Yong Wang, Zhen-Yong Keck, Steven K. H. Foung

**Affiliations:** Department of Pathology, School of Medicine, Stanford University, Stanford, CA 94305, USA; E-Mails: yongw1@stanford.edu(Y.W.); zkeck@stanford.edu (Z.-Y.K.)

**Keywords:** Hepatitis C virus, neutralization antibodies, escape, epitope, vaccine development

## Abstract

A critical first step in a “rational vaccine design” approach for hepatitis C virus (HCV) is to identify the most relevant mechanisms of immune protection. Emerging evidence provides support for a protective role of virus neutralizing antibodies, and the ability of the B cell response to modify the course of acute HCV infection. This has been made possible by the development of *in vitro* cell culture models, based on HCV retroviral pseudotype particles expressing E1E2 and infectious cell culture-derived HCV virions, and small animal models that are robust tools in studies of antibody-mediated virus neutralization. This review is focused on the immunogenic determinants on the E2 glycoprotein mediating virus neutralization and the pathways in which the virus is able to escape from immune containment. Encouraging findings from recent studies provide support for the existence of broadly neutralization antibodies that are not associated with virus escape. The identification of conserved epitopes mediating virus neutralization that are not associated with virus escape will facilitate the design of a vaccine immunogen capable of eliciting broadly neutralizing antibodies against this highly diverse virus.

## 1. Introduction

Up to 170 million people worldwide are chronically infected with hepatitis C virus (HCV), with many at significant risk for cirrhosis, liver failure and hepatocellular carcinoma [1]. The virus is transmitted primarily by parenteral routes. From unsafe needle injections alone, the World Health Organization estimates an annual increase in the global burden by 2 million new infections [2]. Until this year, treatment was primarily with pegylated-interferon and ribavirin, which helped some patients with chronic HCV infection. But viral replication was incompletely inhibited and a high relapse rate occurred when patients discontinued treatment. The recent advances in *in vitro* and *in vivo* HCV infection systems and increased understanding of HCV virology have led to the development of many HCV-specific small molecules with antiviral activity. This has led to new optimism in HCV treatment programs with the recent completion of Phase III studies of several protease inhibitors having promising results [3]. However, the potential for drug resistant HCV mutants from these direct-acting antivirals is an ongoing concern [4]. A vaccine-based preventive strategy is clearly needed. A critical first step in a “rational vaccine design” approach for HCV is to identify mechanisms of immune protection. The induction of neutralizing antibodies following vaccination provides a first line of adaptive immune defense against a number of viral pathogens. For HCV, emerging evidence indicates a protective role of virus neutralizing antibodies, and the ability of the B cell response to modify the course of infection.

## 2. The Role of Neutralizing Antibodies in Controlling HCV Infection

Virus neutralizing antibodies are an important component of protection against many viral pathogens. But for some viruses, such as HCV, the protective role for neutralizing antibodies is not readily apparent because the drop in peak viral load during acute infection is more temporally correlated with cellular immunity rather than the appearance of neutralizing antibodies [5,6]. CD4+ and CD8+ T cell responses correlate with control of acute infection, although they are not sufficient to prevent chronic infection and disease progression [7]. The role of antibodies in preventing and controlling HCV infection has been less defined because of difficulties in having efficient and reliable *in vitro* systems to grow HCV. Nonetheless, clinical trials with IgG therapy before the isolation of HCV demonstrated prevention of transfusion-associated non-A, non-B hepatitis that was due mostly to HCV [8–11]. Other clinical studies showed a reduction of infection transmission among sex partners of HCV-infected patients who received gammaglobulin [12]. Early animal studies supported this observation. An infectious inoculum obtained during acute infection from a patient who eventually developed chronic HCV hepatitis could be neutralized by *in vitro* incubation with plasma of the same subject collected from 2 years after infection [13]. Other early animal studies observed a delay in the onset of acute infection when chimpanzees were treated with HCV gammaglobulin prior to challenge with infectious virus [14].

The development of *in vitro* cell culture models and neutralization assays, based on HCV retroviral pseudotype particles expressing E1E2 (HCVpp) and infectious cell culture-derived HCV virions (HCVcc), has facilitated the measurement of antibody-mediated virus neutralization, and thus to evaluate the impact of antibody in the control of infection [15–19]. This led to the observation that the protective effect provided by IgG preparations in chimpanzee challenged studies correlated with antibody titers blocking infection of target cells with HCVpp [20]. Moreover, studies with retroviral HCVpp have shown a relationship between the control of virus infection and a neutralizing antibody response in single source outbreaks of acute HCV infections [21,22]. One group involved a large cohort of pregnant women who received anti-RhD gammaglobulin contaminated with a single 1b HCV strain, and the other involved hemodialysis patients with nosocomial acquired HCV infection. Both studies, employing HCVpp, showed a relationship between the control of virus infection and the neutralizing antibody response in acute HCV infections [21,22]. Individuals with a strong and progressive neutralizing HCVpp antibody response demonstrated decreasing viremia. Viral clearance was associated with a rapid induction of neutralizing antibodies in the early phase of infection with some evidence that these antibodies are broadly reactive [21,22]. In contrast, chronic HCV infection was characterized by absent or low-titer neutralizing antibodies in the early phase of infection and the persistence of infection despite the induction of cross-neutralizing antibodies in the late phase of infection. Moreover, escape from neutralizing antibodies appears to influence HCV re-infection of the liver graft during liver transplantation [23]. These findings suggest that the quality and quantity of the neutralizing antibody response during acute infection are potential determinants of infection outcome.

Recently developed small animal models have provided additional tools to evaluate the role of antibodies in HCV infection and immunity. One model is based on immunodeficient mice re-populated with human liver fragments [24]. In a homologous *in vivo* protection study, chimeric mice were prophylactically treated with chronic phase polyclonal IgG prepared from serum of an individual with chronic HCV infection. The mice were then challenged with infectious virus obtained from the same individual during acute infection. Infection was prevented in five of eight challenged animals. For the non-protected animals, the HCV infection was attenuated [25]. In a study of a human monoclonal antibody (HMAb) that neutralized genetically diverse genotype HCVpp, prophylactic treatment provided protection against heterologous infectious HCV challenge in this human liver chimeric mouse model [26]. A more recently developed immunocompetent humanized mouse model for HCV exhibited a robust antibody response to vaccination with a recombinant vaccinia virus expressing HCV C-E1-E2-p7-NS2 proteins. Some of the vaccinated animals were protected against a heterologous infectious HCV challenge and protection was correlated with the serum level of antibodies to E2 [27].

The HCV envelope glycoproteins, E1 and E2, are the natural targets of the protective antibody response. The focus of this review is on the immunogenic determinants on E2 mediating virus neutralization. Reported E1-specific neutralizing antibodies are more limited [28,29] and probably reflect that this protein is of lower immunogenicity than E2. However, there is evidence that E1-specific responses can be invoked following experimental vaccination and these responses may be protective [30].

## 3. Neutralizing Antibodies to Linear Epitopes

HCV entry into target cells requires attachment to surface glycosaminoglycans and perhaps lipoprotein receptors leading to the interaction of viral glycoproteins with co-receptors, CD81 and SR-BI. Subsequent to these events, Claudin-I, other claudin molecules, and another hepatocyte tight junction protein, Occludin, are felt to be involved in the entry pathway [31–36]. The envelope glycoproteins, E1E2, are responsible for virus attachment and receptor-mediated endocytosis. However, there is convincing evidence of E2 binding only to CD81 and SR-B1 [37,38]. Monoclonal or polyclonal antibodies targeting both linear and conformational epitopes of E2 have been shown to mediate virus neutralization.

A major determinant of neutralization is the “hyper-variable region 1” (HVR1), which encompass the first 27 amino acids (aa 384–410) located at the N-terminus of HCV E2 [13,39]. This E2 segment is highly immunogenic and antibodies against HVR1 can be detected in the majority of HCV infected individuals [40–42]. Animals immunized with synthetic HVR1 peptide elicit high titer serum antibodies to HVR1 [43,44]. Comparing the protective effect of HVR1 peptide with HCV E1, E2 recombinant protein immunization, protection was more associated with anti-HVR1 antibody titers than with anti-E1 or anti-E2 [45–47]. However, antibodies to HVR1 over time drive replication of viral variants that the existing antibody response does not recognize [13,48,49]. The limited nature of the B cell response to this region is shown in a study of sequential HCV sequences isolated from one patient over a 26-year period [50]. Patient-specific HCVpp expressing sequential envelope variants were employed to assess virus neutralization by autologous sera. While capable of neutralizing earlier quasispecies, serum antibodies failed to neutralize the concurrent dominant HCV E1E2 species that were present in the blood. Escape was associated with mutations within HVR1 leading to decreased binding and neutralization by monoclonal antibodies that were produced to the earliest E2 HVR1 sequence obtained from this patient. Another study of individuals progressing from the acute phase of infection to chronicity confirmed both the relationship between neutralizing antibodies and virus clearance, and escape from neutralization secondary to HVR1 variants [51]. High-titer serum neutralizing antibodies were detected in individuals with spontaneous resolution of infection, peaking at the time of viral clearance, while most individuals progressing to chronic infection demonstrated low-titer or absent neutralizing antibodies throughout early acute infection. When patient-specific HCVpp expressing sequential envelope variants were used to assess neutralization by autologous sera, neutralization of earlier sequence variants was detected prior to later variants, indicating clearance and evolution of quasispecies variants in response to pressure from neutralizing antibodies. Site directed mutagenesis of the pseudotyped envelope sequence revealed amino acid substitutions within HVR1 that were responsible for the loss of neutralization sensitivity over time. One interpretation of these findings is that neutralizing antibodies directed at more conserved epitopes outside of HVR1 are likely to occur late in the course of disease progression, since the majority of infected individuals develop chronic infection. Nonetheless, for some patients, the early appearance of neutralizing antibodies appeared to contribute to the control of viremia. Antibodies to HVR1 are mostly isolate-specific, although there are reports of broadly reactive HVR1 antibodies [40,43,52,53]. Interestingly, delta HVR1 viral particles are more sensitive to antibody-mediated neutralization, which indicates that HVR1 partly shields more conserved epitopes mediating neutralization [54,55]. Taken together, these findings suggest that the HVR1 region encodes immunodominant epitopes that elicit neutralizing antibodies, and in the early acute infection these antibodies have a possible role in the resolution of acute infection. However, these epitopes tend to be isolate-specific and therefore are of limited utility in vaccine development.

**Figure 1.**
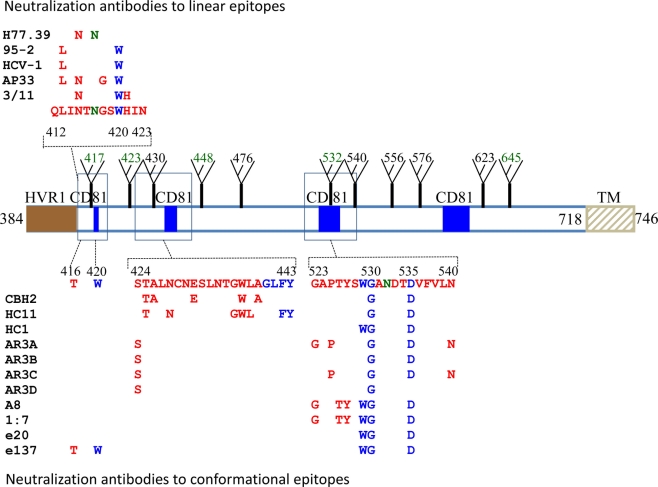
Mapping of neutralization epitopes on the HCV E2 protein sequence. The alignment of the HCV gene sequence is based on the genotype 1a, H77c (GenBank accession no. AF011751). Regions that are associated with CD81 binding (blue square), HVR1 (solid brown square), transmembrane domain (TM) and glycosylation sites (fork) are as indicated. Contact residues broadly neutralizing antibodies to both linear and conformational epitopes are as listed. Residues that participate in CD81 binding are labeled in blue and glycosylation sites that affect antibody neutralization are in dark green. For the conformational epitopes, contact residues are located mainly at two discontinuous regions, aa424–443 and aa523–540.

**Figure 2.**
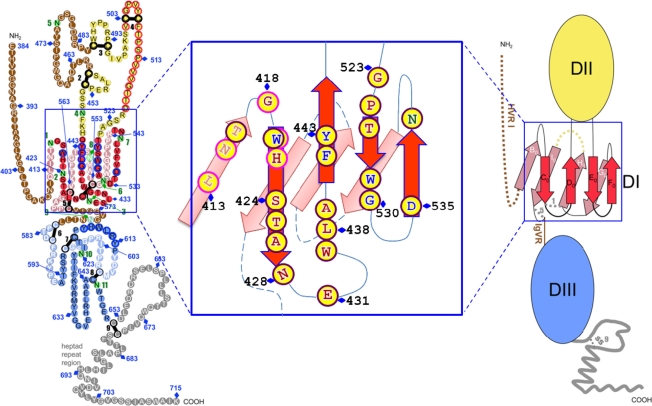
Putative model of HCV E2 glycoprotein based on a class II fold with the expansion of Domain I and the contact residues recognized by broad neutralization antibody. The linear sequence of HCV H77-E2 ectodomain from aa384–715, is represented as a chain of beads (right) or schematic diagram of the tertiary organization (left), and the expanded DI is shown in the middle. For the model on the right, colored circles are labeled with the corresponding amino acid. Circles in pale and bright colors represent residues in the background and foreground of the domains, respectively labeled in white and black fonts. Residues that participate in CD81 binding are contoured in blue. Disulfide bonds and glycosylation sites are indicated by thick black bars and green circles, respectively, numbered sequentially. Unstructured segments are in white font on a brown background. For the left schematic diagram, DI, DII and DIII are labeled in red, yellow and blue, respectively. The connectivity of the β-strands in DI is indicated, labeled with the standard class II nomenclature. In the middle graph, the conserved epitopes recognized by broadly neutralization antibodies are labeled with yellow circle in the expanded DI. The residues recognized by linear epitope are outlined with bright purple, while conformational epitopes are outlined with dark purple. Residues that participate in CD81 binding are labeled in blue and glycosylation sites in dark green. Some of the residues are numbered as the amino acid position in the E2 glycoprotein. The specific contact residues for the conformational antibodies are located on four β-strands, C_0_, D_0_, E_0_ and F_0_, which form the top β-sheet of domain I, overlapping with CD81 binding residues. Figure 2 is adapted with permission from Krey *et al.* [62].

An E2 segment that is adjacent to HVR1, encompassing aa410–425, is recognized as encoding highly conserved neutralizing epitopes. The mouse monoclonal antibody, AP33, was the first reported antibody that defined a linear epitope in this region having contact residues within aa412–423 [56,57]. The antibody displays broad neutralization against HCVpp bearing E1E2 representatives of the major HCV genotypes 1 through 6 [57], which is consistent with this epitope being highly conserved. Other monoclonal antibodies of both murine and human origins, such as 3/11, 95-2 and HCV-1, have been reported to bind to epitopes within aa412–423 and displaying broadly neutralizing activities [57–59]. Epitope mapping showed that the W420 is a conserved contact residue shared by these antibodies (Figure 1). W420 also serves as a critical residue for virus binding to CD81 [60]. Another murine monoclonal antibody, H77.39, has been described to bind to an epitope containing contact residues within aa410–425, at N415 and N417 [61]. Interestingly, this antibody inhibits both E2 binding to CD81 and SR-B1. In a proposed model of the tertiary organization of HCV E2 [62], the AP33 epitope is comprised of contact residues located on two β-strands, B_0_ and C_0_ within domain I (Figure 2) (see [63] for domain and strand nomenclature). The contact residue for The AP33 epitope is mostly regarded as a linear epitope, but it contains a conformational component, as suggested by a modest reduction in AP33 binding to denatured E1E2 [56]. This was confirmed by *in vitro* selection of AP33 neutralization-escape virus mutants that located an escape mutation at a considerable distance from aa412–423 [64]. Unlike HVR1, antibodies to the aa412–423 appear to be less prevalent and present in less than 2.5% of sera obtained from individuals with chronic HCV infection, and in 1 from 32 patients who resolved HCV infection. This finding suggests that AP33-like antibodies do not play a major role in natural clearance of HCV infection [65]. The 412–423 epitope also appears to be weakly immunogenic in the HuMAb mice (transgenic mice containing human antibody genes) immunized with soluble E2. Of the total 51 immunized mice, hybridomas producing antibodies reactive to this epitope were isolated from only two animals [58].

Analysis of over 5,500 sequences obtained from GenBank database showed that the AP33 epitope is highly conserved. In spite of this observation, escape mutations within aa412–423 have been documented. In the presence of AP33, a N415Y mutant arose under selection pressure. The mutant variant is resistant to AP33-mediated neutralization but the mutation is associated with a cost in virus fitness [64]. Collectively, this suggests that the AP33 epitope should be incorporated in an HCV vaccine. However, multiple mutations, G418D and N415D, have been reported that escape AP33-mediated neutralization and not associated with a lowering of virus fitness. Of note is that these mutations contributed to AP33-escape variants that were more sensitive to neutralization by other neutralizing HMAbs to E2 [66].

## 4. Neutralizing Antibodies to Conformational Epitopes

The majority of antibodies with broad neutralizing activities recognized conformational epitopes on E2 [26,67–70]. In a panel of HCV HMAbs generated from peripheral B cells of patients with chronic HCV infection, cross-competition analyses delineated at least three immunogenic clusters of overlapping epitopes with distinct properties [71,72]. Non-neutralizing HMAbs fell within one cluster, which we have designated as “domain A”, while neutralizing HMAbs segregated into two clusters, designated as domain B and C. HMAbs to domain B display varying neutralizing activity against HCVpp containing glycoproteins of HCV genotypes 1 to 6, and some neutralizing all genotypes, such as the antibody designated as CBH-5 [73,74]. Although HC-1 has a broad neutralization profile, the potency against most HCV isolates was modest [73,74]. After affinity maturation by random mutagenesis on yeast surface display, affinity-matured HC-1 IgG clones showed improved neutralizing potency against a panel of genotype 1–5 HCVpp isolates and 2a HCVcc [75]. Of note, some of the affinity-matured antibodies neutralized a viral isolate that was not neutralized by wild-type HC-1 [75]. Alanine substitution studies revealed that two conserved E2 residues, G530 and D535, are required for binding of all domain B HMAbs, while G523 or W529 are required for some but not all of these antibodies [73,74]. Importantly, W529, G530 and D535 participate in the interaction of E2 with CD81. The data thus suggest that domain B HMAbs exert potent and broad neutralizing effects on HCV by competing with CD81 for binding to conserved residues on E2 that are important for viral entry. For certain domain B antibodies, including CBH-2 and HC-11, epitope mapping also revealed contact residues within the region encompassing aa425–443 (Figure 1) [76]. Residues within aa425–443 have been implicated in E2 binding to CD81 [77]. Consistent with this, studies by multiple groups on broadly neutralizing HMAbs isolated from combinatorial libraries also recognized epitopes containing these residues [26,78,79]. In one study, their antibodies were sorted into three groups, designated as AR1, AR2 and AR3, based on different binding patterns and competition with other defined monoclonal antibodies. While AR1 and AR2 antibodies, had limited breadth of neutralization, AR3 antibodies showed broad neutralization against genotype 1–6 HCVpp. In addition, an AR3-specific antibody protected Alb-uPA/SCID mice against the diversity of HCV quasispecies in an infectious inoculum. Epitope mapping identified AR3 epitopes being formed by three discontinuous E2 segments, aa394–424, aa437–447 and aa523–540. Alanine scanning revealed contact residues located at S424, G523, G530, D535, V538 and N540 (Figure 1) [80]. Broadly neutralizing HMAbs, including A8, 1:7, e137 and e20, reported by other group also mapped to the E2 segment, aa523–540, and identified the same conserved contact residues located at W529, G530 or D535 (Figure 1) [78,79,81].

In addition, in the proposed model of the tertiary organization of HCV E2 [62], the residues in the CD81 binding site on HCV E2 lie in domain I, which is a β-sandwich formed from the C_0_D_0_E_0_F_0_ (top) and the B_0_I_0_H_0_G_0_ (bottom) β-sheets. The binding site of these antibodies maps to the exposed top β-sheet (Figure 2). The β-strands forming the top sheet are segregated into two contiguous sequences, aa418–444 for C_0_D_0_ and aa526–542 for E_0_F_0_. The location of the contact residues for each of the neutralizing antibodies to conformational epitopes are also on two discontinuous E2 segments encompassing aa425–443 and aa523–540 (Figure 1), which are respectively contained in C_0_D_0_ and E_0_F_0_, and are in in close proximity within the top β-sheet. Thus, the location of these contact residues provides support for the model and indicate that these antibodies bind to the same exposed tertiary structure on E2 that interacts with CD81 (Figure 2).

Understanding the interaction between broadly neutralizing antibodies and their epitopes provides the basis for rational design of a preventive HCV vaccine. The successful isolation of these antibodies suggests that domain B is a highly immunogenic region on HCV E2 containing a cluster of overlapping epitopes that are able to induce potent neutralizing antibodies. This is supported by the observation that an HCVpp mutant with an alanine substitution at N532A exhibited greater sensitivity to neutralizing sera obtained from individuals infected with HCV genotype 1a, 1b, 2b, 3, 4 or 5 viruses [82], which is consistent with these sera contain domain B-like antibodies directed at residues near N532. Two questions are of concern from a vaccine perspective: which of the domain B epitopes are prone to accumulating mutations under immune pressure, leading to virus escape from neutralization, as observed with the antibody response to HVR-1 and aa412–423; and, which domain B epitopes remain relatively invariant so as to accommodate the interactions of E2 with CD81 required for virus viability. CBH-2, HC-11 and HC-1 are representatives of antibodies to overlapping epitopes on E2 mediating neutralization by blocking virus binding to CD81 (Figure 1). In a study with an approach that allowed escape variants to be amplified and isolated under increasing concentrations of a neutralizing antibody, three escape patterns were observed with these antibodies. For CBH-2, escape mutants were isolated containing mutations at D431G or A439E [76]. The induction of the escape mutant at D431G under the selective pressure of CBH-2 mimics the previously observed naturally occurring variant at this site, D431E, that was not neutralized by this antibody [83]. Furthermore, the similarity of the replication rate of infectious CBH-2 escape HCVcc mutants to that of wt HCVcc suggests that escape from CBH-2-like antibodies does not compromise viral fitness. In the escape studies with HMAb HC-11, wt HCVcc was completely eliminated, and no escape mutants were generated under a starting antibody concentration of 100 μg/mL. However, under the condition of increasing antibody concentrations starting at 0.05 μg/mL, an escape mutant with a single substitution at L438F was observed when the antibody concentration reached 10 μg/mL. A mutant containing double substitutions, located at L438F and N434D, appeared after continuing passage of the virus at selection 10 μg/mL. Another mutant with double substitutions at L438F and T435A appeared when HC-11 was increased to 100 μg/mL. By gradually increasing the selection pressure by gradually increasing the concentration of the antibody, escape mutants were isolated that were not apparent when infectious virus encounters a high antibody concentration. The implication for vaccine design is a requirement for the immunogen, containing an HC-11-like epitope, to be able to elicit a sufficiently high titer antibody response to avoid virus escape. If the antibody response is of low titer, virus escape can occur. Nonetheless, the escape mutations resulted in a progressive decrease in viral fitness under the selective pressure of HC-11. Huh7.5 cells infected with L438F HC-11 escape mutant at a low multiplicity of infection (MOI = 0.01) yielded eight times less virus than wt HCVcc. The double substitution mutant, L438F and T435A, yielded 256 times less virus. For HC-1, the antibody at a critical concentration completely suppressed viral replication and no escape mutants were isolated. The location of the contact residues for HC-1 appears to be limited to a more invariant segment of E2 encompassing aa523–540 that interacts with CD81. Collectively, these findings highlight the substantial challenges inherent in developing HCV vaccines, and show that an effective vaccine will need to induce antibodies to intrinsically conserved epitopes in order to lessen the probability of virus escape.

## 5. Negative Modulation of Neutralizing Antibodies

In addition to genetic mutation escape strategy from neutralization, there are other evasion strategies to negatively modulate the neutralizing antibody response. Glycosylation of the virus envelope, non-neutralizing antibodies or virion-associated lipoproteins may interfere with antibody-mediated neutralization by masking neutralizing epitopes or otherwise limiting access of neutralizing antibodies to their cognate epitopes [82,84,85]. Studies with HCVpp and HCVcc indicate that N-linked glycans at conserved Asn residues of the E2 protein hinder neutralizing activities of HCV-specific polyclonal sera, as well as neutralizing HMAbs [82,84,86]. The fine mapping of domain B HMAbs provides an exact explanation why specific N-glycan sites on E2 may modulate antibody-mediated neutralization [82,84]. The N-glycan at Asn532 sits in the middle of two critical contact residues for all domain B antibodies and is therefore capable of hindering antibody binding. The substitution of the N-glycan at residue 532 could lead to an improved HCV immunogen. For non-neutralizing antibodies, it has been proposed that a segment of E2 encompassing aa434–446 (epitope II) encodes a cluster of epitopes that are associated with non-neutralizing antibodies, and that these antibodies inhibit the neutralizing activities of antibodies directed at an adjacent E2 segment encompassing aa412–426 (epitope I) [87,88]. Depletion of the epitope-II antibodies in polyclonal IgG preps led to increase neutralizing titers of these antibody preparations [88]. If this is the case, it provides a contributing factor in explaining the existence of viremia in the context of high neutralizing antibodies titers in chronic HCV infection. The circulating HCV virions in the blood of patients, associated with host lipoproteins and immunoglobulin in the form of lipo-viro-particles (LVP), are heterogeneous in density and infectivity. The LVP corresponding to low density fractions expressed high infectivity, which could be blocked by the anti-apolipoprotein B (Apo B) and E antibodies [89]. HCV envelope glycoproteins have the intrinsic capacity to utilize lipoprotein synthesis and assembly machinery, and HCV virions production is closely connected to lipid metabolism which could serve as an assembly platform [90–92]. The extent to which the association of HCV with lipoproteins may impede access to the virion surface by neutralizing antibodies is not clear. Infectious HCVcc particles can be readily precipitated with antibody to ApoB, but are nonetheless neutralized with virus-specific HMAbs [93]. In the presence of high density lipoprotein (HDL) or human serum, the neutralization by anti-E2 neutralizing antibodies of HCVpp and HCVcc could be attenuated [94]. Of HCV particles recovered from the liver of an immunodeficient patient, only 27% of the E2 and 8.5% of RNA could be immunoprecipitated with AP33 [95]. In contrast, two of our domain B HMAbs precipitated 52–67% of total E2 and 19–24% of total RNA [95]. Clearly, not all neutralizing epitopes are masked by lipids, but better quantitation of this potential negative regulator of neutralizing activity will require additional studies of wild-type HCV.

In addition to virus entry by receptor-mediated endocytosis, HCV has been reported to spread from cell-to-cell [96–100]. This process appears to be mediated by HCV receptor molecules, CD81, SR-B1, Claudin-1 and Occludin [96,98], although there is some controversy whether CD81 is involved in this process [97]. While initial studies showed that neutralizing antibodies do not inhibit cell-to-cell spread, there is some evidence that antibodies to SR-B1 and to HVR1 do inhibit this route of transmission [96,98]. Moreover, a small molecule inhibitor of HCV entry that binds to the carboxy-terminal region of E2 also inhibits cell-to-cell spread [99]. Taken together, the implication is that some antibodies to E2 could be effective in blocking cell-to-cell spread.

## 6. Conclusions

These studies illustrate the complexity of the antibody response to HCV and the multiple aspects of the ability of the virus to escape from antibody-mediated virus neutralization. While candidate neutralizing antibodies to highly conserved conformational and linear epitopes have been identified, a greater understanding of the factors contributing to virus escape or interference with virus neutralization will be required to define those protective determinants most likely to provide broad protection. Additional studies will also be required on the possible role of lipoproteins in masking virion surface domains involved in virus entry, although current data suggest that a specific cluster of epitopes (designated domain B) on HCV E2 remains exposed on low density cell culture infectious HCV virions. The emergence of escape viral mutants may be slowed by the successful induction of neutralizing antibodies to multiple determinants in an HCV vaccine. The encouraging data from these studies proved the existence of broadly neutralization antibodies that are not associated with virus escape. The identification of conserved epitopes shared by broadly protective antibodies, especially those that lessen the probability of virus escape, suggest that active immunization strategies can in fact be a realistic goal.
